# Supplementary material for: Causal Relationship between Aging and Anorexia Nervosa: A White-Matter-Microstructure-Mediated Mendelian Randomization Analysis
Source: Biomedicines. 2024 Aug 16;12(8):1874. doi: 10.3390/biomedicines12081874 (PMC11351342; doi:10.3390/biomedicines12081874)
Supplement: Supplementary file 1 [file biomedicines-12-01874-s001.zip › Supplementary Figure/Figure S8/Leaveoneout plot of MR analysis for TL to ubm-b-1569.pdf]

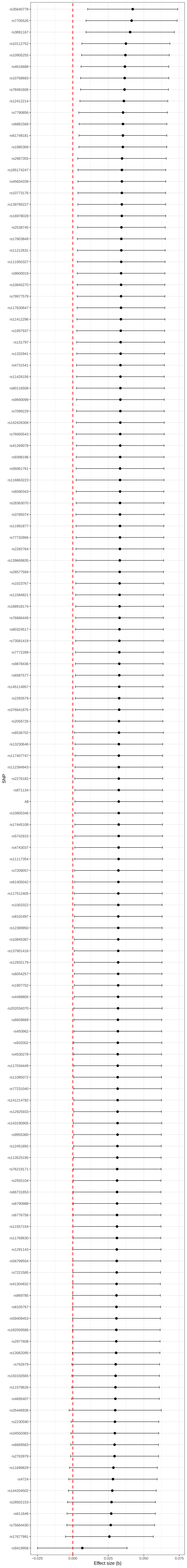
rs35640778
rs7705526
rs3891167
rs10112752
rs10905255
rs4616688
rs10768683
rs78491606
rs12412214
rs7790856
rs6881568
rs61748181
rs1985369
rs2967355
rs185174247
rs45604339
rs10773176
rs139795227
rs16978028
rs2538745
rs17803849
rs11212631
rs111950327
rs9600019
rs10840270
rs79977579
rs117630647
rs11412296
rs1957937
rs131797
rs1332941
rs4731541
rs11426156
rs80116508
rs9940099
rs7099229
rs142426306
rs76065543
rs41269079
rs9398196
rs56061761
rs116863223
rs6590343
rs28363070
rs3785074
rs11991877
rs77732866
rs2282764
rs139669835
rs28577594
rs1023767
rs11584821
rs188918174
rs76664449
rs80324517
rs73581419
rs7772289
rs9878436
rs6587577
rs145114957
rs2293579
rs376641875
rs2056726
rs6536702
rs13230646
rs117407747
rs112394943
rs2276182
rs871134
All
rs10805346
rs17445108
rs5742915
rs4743037
rs11117354
rs7209057
rs61405042
rs117512405
rs1003322
rs8102497
rs12369950
rs10845387
rs137901416
rs12932179
rs6054257
rs1907702
rs4498805
rs202034370
rs6659669
rs450962
rs932002
rs4530278
rs117034449
rs11085072
rs77231040
rs141214782
rs12925933
rs143190905
rs9955360
rs12451892
rs113525105
rs76219171
rs2555104
rs66731853
rs6790988
rs6776756
rs11557154
rs11769630
rs1291143
rs56799554
rs7221585
rs41304832
rs869785
rs8105767
rs59409453
rs182059586
rs2977608
rs13062095
rs762679
rs150150565
rs11579626
rs4695407
rs35446936
rs2230590
rs34550383
rs6669563
rs2763979
rs11699829
rs4724
rs144204502
rs28502153
rs611646
rs75664430
rs17677991
rs9419958
SNP
-0.025
0.000
0.025
0.050
0.075
Effect size (b)
